# A systematic review and meta-analysis of the efficacy of organic acids in reducing *Salmonella* colonization in the crop and ceca of broilers

**DOI:** 10.1016/j.psj.2025.106075

**Published:** 2025-11-07

**Authors:** J. Wang, B. Mallavarapu, D. Subedi, P.S. Patil, S. Bhumanapalli, S. Vaddu, A.K. Singh, W.K. Kim, S. Kumar, S. Poudel, D.V. Bourassa, S. Manjankattil, M. Naeem, Y. Adhikari, H. Thippareddi

**Affiliations:** a Department of Poultry Science, Auburn University; b Department of Poultry Science, University of Georgia; c Department of Agriculture and Natural Resources, Delaware State University; d Niagara Bottling, Diamond Bar, CA

**Keywords:** *Salmonella*, Systematic review, Meta-analysis, Broiler, Food safety, Organic acid

## Abstract

Organic acids have been widely used as feed and water supplements during broiler grow-out and feed withdrawal to reduce *Salmonella* colonization, with variable efficacy. This systematic review and meta-analysis evaluated the efficacy of supplementing organic acids delivery route (water or feed) on the prevalence and concentrations of *Salmonella* in the crop and ceca of broilers. A total of 2,290 identified publications were screened, 22 (prevalence) and 25 (concentration) publications met inclusion criteria. Mean values, standard deviations, and replicates for control and treatment groups were extracted to calculate the mean difference (log CFU) from studies reported concentration results. The number of positives and sample size were extracted to calculate the odds ratio from studies reporting prevalence results. Data were analyzed using meta package in R (α = 0.1). All models had a medium or high heterogeneity (*I^2^* > 50 %), therefore, results from the random effect model were reported. Meta-analysis revealed that supplementing organic acids via feed reduced the odds of *Salmonella* positives in the crop by 84 % compared to the control (*P* < 0.01), while supplementation through drinking water was less effective, reducing the odds by 57 % compared with the control (*P* < 0.01). Both water and feed delivery routes reduced the odds of *Salmonella* positives in the ceca by 62 % and 66 %, respectively (*P* < 0.01). Organic acid supplementation in the drinking water and feed was estimated to reduce *Salmonella* concentration in the crop by 0.70 (*P* < 0.01) and 0.92 log CFU (*P* = 0.062), respectively. Similarly, both routes are estimated to reduce *Salmonella* concentration in the ceca by 0.72 log CFU from water and 1.59 log CFU from feed, respectively (*P* < 0.01). Organic acid supplementation effectively reduces *Salmonella* in broilers at pre-harvest, with feed delivery generally showing greater efficacy for prevalence reduction. However, caution should be exercised when incorporating organic acids, and their optimal dosage and safety must be evaluated before implementation.

## Introduction

*Salmonella* is the leading cause of foodborne illness in the U.S., responsible for an estimated 1.03 million infections and $4.1 billion in annual direct medical costs (Marshall et al., 2024). The consumption of contaminated poultry products is the primary source of non-typhoidal salmonellosis, accounting for over 24 % of U.S. cases (NACMCF, 2024). The USDA FSIS investigated a total of 52 meat and poultry outbreaks, with 31 outbreaks attributed to *Salmonella*, and 15 specifically linked to chicken and turkey products (GAO, 2025). The USDA FSIS published pathogen performance standards for raw chicken parts, comminuted chicken, and comminuted turkey to reduce salmonellosis risk from consumption of poultry products (USDA FSIS, 2016). While antimicrobial interventions at poultry processing have decreased *Salmonella* prevalence on raw chicken by 75 % (IFSAC, 2022), it remains a significant food safety concern. The US CDC reported a salmonellosis incidence rate of 15 cases per 100,000 population attributed to chicken (Tack et al., 2019). The USDA FSIS published ‘Proposed Regulatory Framework to Reduce *Salmonella* Illnesses Attributable to Poultry’ a comprehensive effort to reduce *Salmonella* illnesses associated with poultry products (USDA FSIS, 2022). Out of three components, the first component in the framework is identifying and implementing antimicrobial intervention strategies at pre-harvest to reduce *Salmonella* entering the poultry slaughter facilities on the surface or in the gastrointestinal tract (GIT) of the birds.

A major challenge for controlling *Salmonella* in poultry products is that broilers and turkeys often harbor pathogens during live production (Golden and Mishra, 2020). Avian GIT and poultry production environment serve as a reservoir for *Salmonella* and *Campylobacter*, directly contributing to carcass contamination during processing (Wang et al., 2023a, b; Adhikari et al., 2024, 2025). Foodborne pathogens enter poultry live production facilities through various vectors, including chicks, contaminated feed or water, reused litter and surrounding environments (Totton et al., 2012). Reducing *Salmonella* load at pre-harvest is therefore critical to minimize contamination of the carcasses and carcass parts at processing (Machado Junior et al., 2020). Various pre-harvest interventions including biosecurity, vaccination, and dietary/ water treatments (e.g., probiotics, organic acids, essential oils) aim to mitigate *Salmonella* colonization in the birds (Obe et al., 2023; Naeem and Bourassa, 2024). However, the efficacy of dietary and water treatments varies significantly due to the challenge dose, serovars, rearing conditions, production system, birds’ age, and genetics etc.

Organic acids are naturally occurring or fermentation derived carboxylic acids with antimicrobial properties and are commonly supplemented through poultry diet and water. Extensive research has been conducted on the evaluation of organic acids and their derivatives on the efficacy of controlling foodborne pathogens, with inconsistent results. For example, some reports (Hernandez-Patlan et al., 2019; Adhikari et al., 2020; Ferreira et al., 2022) indicated that incorporation of organic acids reduced *Salmonella* prevalence and its concentration in ceca or litter during broiler production, while others reported lack of efficacy. This variability may stem from differences in acid type, concentration, delivery route, or production environment. Therefore, the meta-analysis and systematic review approach becomes a valuable tool to draw conclusions with greater statistical power.

This study evaluated the efficacy and consistency of organic acid supplementation of the feed and/or water on *Salmonella* prevalence and concentration in broilers at pre-harvest. Since organic acids supplementation is through feed or water in poultry production, the emphasis was given to evaluating the effects on different sections of the poultry gut, the crop and the ceca.

## Materials and methods

### Literature search

A systematic review and meta-analysis were conducted following the Preferred Reporting Items for Systematic Reviews and Meta-Analyses (**PRISMA**) guidelines to address: “Does organic acids application in poultry production reduce the concentration and/or prevalence of *Salmonella* in the gastrointestinal tract of meat-type poultry at pre-harvest?” A literature search was performed with the following search terms: (poultry OR chicken OR turkey) AND (*Salmonella*) AND (concentration* OR prevalence* OR colonization*) AND (crop* OR ceca* OR excreta* OR cloaca* OR litter*) and (organic acids* OR short chain fatty acid* OR medium chain fatty acid* OR glyceride*) in the databases Web of Science, PubMed and Google Scholar from January 2000 to December 2024. A total of 2,290 studies were retrieved from PubMed, Scopus, Web of Science, and Google Scholar. One additional study was identified from an article reference. Peer-reviewed articles, conference proceedings, theses, and dissertations were included. No language limitation was set at this step.

### Inclusion criteria

The title and abstract of each article were reviewed for their eligibility to be included in addressing the proposed research question. Inclusion criteria used were: 1) English language; 2) *in vivo* or poultry experiments; 3) organic acids or any derivatives (salt form or glycerides) in poultry production at pre-harvest; 3) test against *Salmonella*. Full text of all potentially eligible studies was collected for detailed review after screening abstracts. Additional eligibility requirements were used while screening the full text. Only the studies reported sufficient data to perform a meta-analysis were included (sample size, mean, and standard deviation or standard error for concentration outcomes; and sample size and number of positives for prevalence outcomes for both the control and treatment groups). Studies with the reputable and replicable microbiological methods were determined to be appropriate to include in the meta-analysis. Disagreements were resolved through consensus between authors.

### Data extraction

Data were extracted and stored using a pre-designed Microsoft Excel spreadsheet. Tabular data were recorded as presented in original publications while graphical data were digitized using ImageJ software (Schindelin et al., 2015). Qualitative data included tested *Salmonella* serotypes, infection method, organic acid type, organs sampled and delivery route. Quantitative data included sample size, mean outcome, and standard deviations of *Salmonella* concentration or sample size and positives for both control and treatment groups. For studies reporting *Salmonella* concentration at multiple time points, the final pre-slaughter measurement was prioritized to reflect intervention efficacy closest to harvest. Quality scores were not assigned to the extracted data in the meta-analysis to prevent any inadvertent selection bias (Stone et al., 2019). For studies that reported multiple intervention doses, the data was pooled using a formula as reported in Chapter 7.7.3.8 and Table 7.7a of the Cochrane Handbook for Systematic Reviews of Interventions (Higgins and Green, 2008).

### Data analysis

The meta-analysis was performed using R software 4.4.2 (R Core Team, 2024), with the meta package (Schwarzer, 2007). For studies reporting *Salmonella* concentration (mean, standard deviation, and sample size for the treatment/control groups), mean differences in log CFU/mL or Log CFU/g (chosen for biological relevance and interpretability of microbial load reduction) and pooled results using the metacont function.

For studies reporting prevalence as a binary outcome, the number of positive samples and the total sample size for both treatment and control groups were extracted. These data were used to compute odds ratios (ORs) to assess the effect of organic acid supplementation on the likelihood of pathogen presence using the formula

$$OR=\frac{a\cdot d}{b\cdot c}$$

Where, *a* represents the number of positive samples in the treatment group, *b* the number of negative samples in the treatment group, *c* the number of positive samples in the control group, and *d* the number of negative samples in the control group.

Prevalence data were pooled using metabin function with inverse-variance weighting (Schwarzer, 2007). In cases where zero-cell counts were present in either the treatment or control group, a continuity correction of 0.5 was applied to all cells of the corresponding 2 × 2 contingency table to enable the calculation of effect size (Sweeting et al., 2004; Leone et al., 2024).

Subgroup analyses of data were performed based on the delivery route (water or feed) of organic acids in crop and ceca. The between-study variance (τ^2^) was estimated using the DerSimonian and Laird method (DerSimonian and Kacker, 2007; Schwarzer et al., 2015). The value of *I^2^* up to 40 % was considered not important, 40–75 % was considered moderate, and beyond 75 % was considered substantial (Deeks et al., 2019).

### Test for publication bias

Due to limited subgroup sizes (*n* < 10) and moderate to substantial heterogeneity (*I*^2^ ≥ 50 %) inherent in animal trials, we could not reliably assess publication bias using funnel plot asymmetry tests. This aligns with Ioannidis and Trikalinos (2007), who noted that such constraints restrict the validity of these tests in meta-analyses.

## Results and discussion

**Fig. 1:**
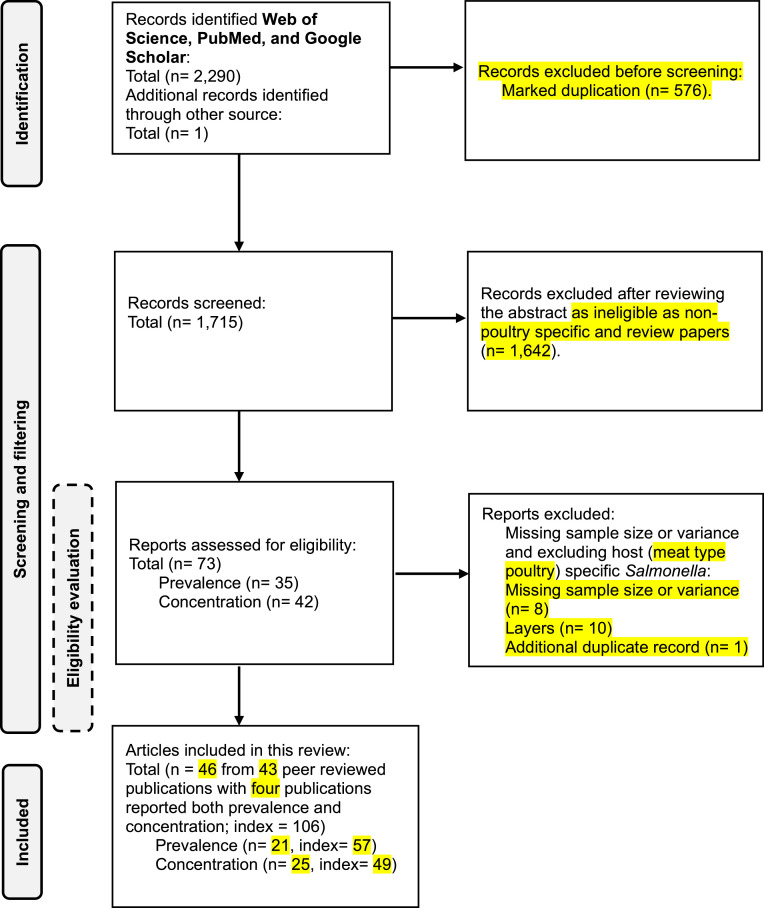
A flowchart showing the outcomes of a literature search done using the Web of Science, PubMed, and Google Scholar databases for systematic review and meta-analysis.

Organic acids and their derivatives are widely incorporated into broilers diet and water as strategies to enhance feed hygiene, improve growth performance, and reduce the colonization risk of enteric pathogens like *Salmonella* (Jansen et al., 2014; de Castro Burbarelli et al., 2017; Polycarpo et al., 2017; Adhikari et al., 2020; Hu et al., 2023). The impact of organic acid supplementation in feed and water on growth performance is well-documented (Polycarpo et al., 2017). This meta-analysis specifically evaluates their efficacy and consistency of reducing *Salmonella* prevalence and concentrations in the poultry GIT. The detail of systematic review process is outlined in Fig. 1.

A total of 2,290 studies were identified through Google Scholar, Web of Science, and PubMed, with one additional study found via manual search. After applying exclusion criteria, 21 and 25 peer-reviewed publications were included in the analyses of *Salmonella* prevalence and concentration, respectively. Among prevalence studies, 17 and 40 interventions examined efficacy in the crop and ceca, respectively. Similarly, among concentration studies, 16 and 33 interventions focused on the crop and ceca, respectively. The characteristics of these studies are summarized in the supplementary material Tables S1 and S2.

### *Salmonella* colonization models

Extensive research has been conducted on the presence of *Salmonella* in live production environments (e.g., boot or drag swabs) and poultry processing facilities (De Villena et al., 2022; Adhikari et al., 2025). However, limited information is available on *Salmonella* prevalence and concentrations in the GIT of birds, particularly in the crop and ceca, during production. Additionally, studies typically use two colonization models, natural colonization from environmental sources (non-challenged) or administration of a *Salmonella* gavage (marker or wild strain). However, the impact of these two colonization models on antimicrobial intervention efficacy in reducing *Salmonella* in the poultry GIT remains unclear. Therefore, this systematic review and meta-analysis first assessed baseline *Salmonella* prevalence and concentration in experimental conditions, as well as the effect of the infection model on pathogen load in the GIT.

**Fig. 2:**
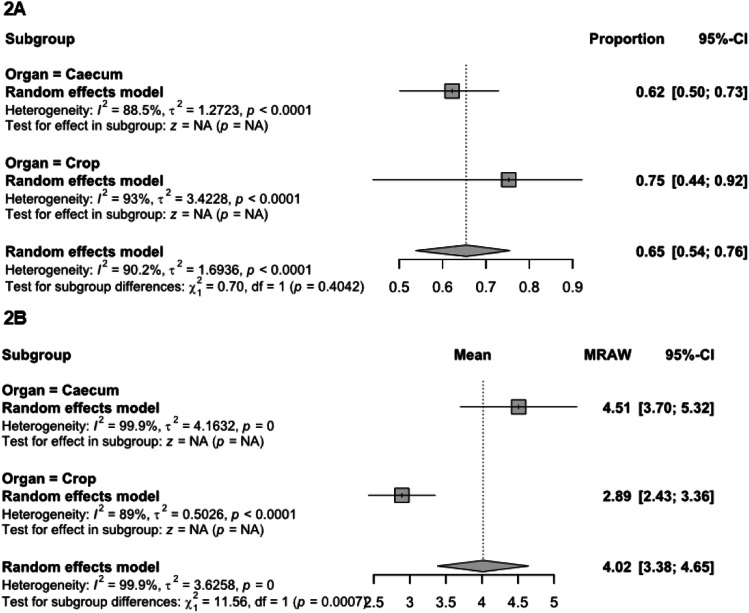
Baseline *Salmonella* prevalence and concentration in control group. Subgroup summary forest plot of subgroup analysis for baseline *Salmonella* prevalence (2A) and concentration (2B) in crop and ceca from the control group. MRAW, mean of raw concentrations in log CFU. Abbreviations: MRAW, mean of raw concentrations in log CFU; CI, confidence interval; τ^2^, tau-squared; χ^2^, chi-squared; df, degrees of freedom; I^2^, I-squared statistic. The vertical dash line in the scale of the forest plot is the line of overall effect.

***Salmonella baseline.*** *Salmonella* prevalence and concentration in the control group, which received no intervention (no organic acid administration), were used to estimate the baseline *Salmonella* load in broilers reared under experimental conditions. Baseline results of *Salmonella* prevalence in the ceca and crop are presented in Fig. 2A. The heterogeneity was substantially high for both the *Salmonella* prevalence and concentration model (*I^2^* = 90 %). Thirty-four studies reported results of *Salmonella* prevalence in the control group, including 8 for crop and 25 for ceca. *Salmonella* prevalence in crop and ceca is estimated to be 75 % (95 % CI: 44 to 92 %) and 62 % (95 % CI: 50 to 73 %), respectively. For the *Salmonella* concentrations, a total of 36 studies from the control group, including 11 in crop and 25 in ceca, were used to estimate the overall *Salmonella* concentration (Fig. 2B.). *Salmonella* concentration in the broiler crop and the ceca is estimated to be 2.89 log CFU (95 % CI: 2.43 to 3.36 log CFU) and 4.51 log CFU (95 % CI: 3.70 to 5.32), respectively, with the population being higher in the ceca (*P* < 0.01).

***Recovery of Salmonella in crop and ceca.*** Both crop and ceca are major colonization sites in the poultry GIT, but their contamination risks differ. While *Salmonella* prevalence in the crop and ceca is comparable (*P* = 0.037), the prevalence in the crop is numerically higher (by 13 %). In controlled experiments, varying results have been reported depending on factors such as bird sampling age, inoculation method, and rearing conditions etc. (Hume et al., 1996; Ramirez et al., 1997; Adhikari et al., 2020; Bourassa et al., 2018; Wang et al., 2024). In commercial poultry production, crop not only harbors higher *Salmonella* prevalence, but it is also more prone to rupture during slaughter, suggesting a greater risk of cross-contamination (Hargis et al., 1995). However, Rostagno et al. (2006) reported a significantly higher *Salmonella* prevalence in the ceca (22 %) compared to the crop (9 %) in a commercial turkey processing facility. Despite these variations, the consistently higher *Salmonella* concentration observed in the ceca (Byrd et al., 2001; Mohyla et al., 2007; Hernandez-Patlan et al., 2019), aligns with the present study and suggests greater risk of cross-contamination risk during poultry processing. These variations linked to bird age, inoculation methods, and rearing conditions warrant further study to quantify their impact on carcass contamination.

**Fig. 3:**
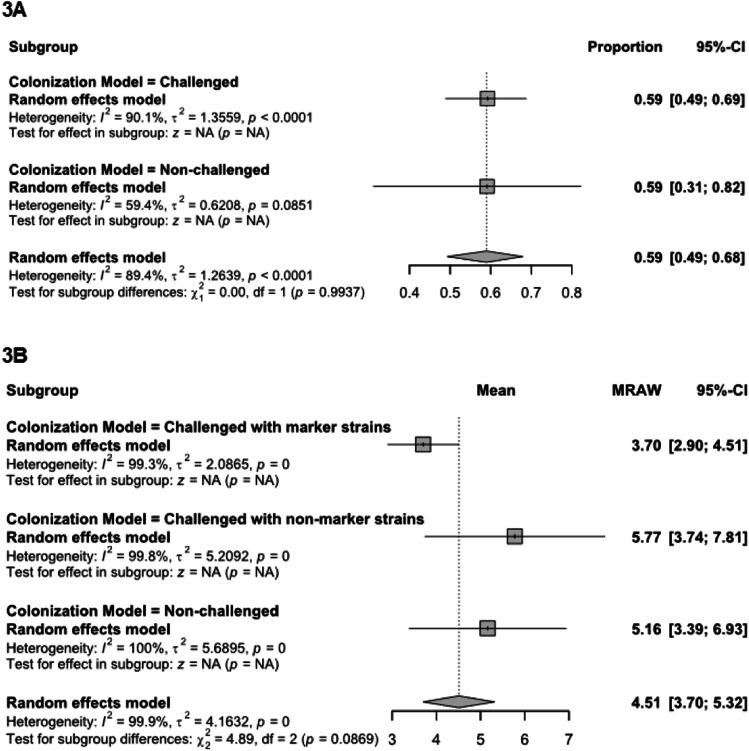
*Salmonella* recovery (prevalence and concentration) from different infection models. Subgroup summary forest plot of subgroup analysis for baseline *Salmonella* prevalence (3A) and concentration (3B) in ceca from the control group across different infection models. Abbreviations: MRAW, mean of raw concentrations in log CFU; CI, confidence interval; τ^2^, tau-squared; χ^2^, chi-squared; df, degrees of freedom; I^2^, I-squared statistic. The vertical dash line in the scale of the forest plot is the line of overall effect.

***Impact of marker strains.*** All studies that reported *Salmonella* prevalence in the crop used the challenged model and consequently, further subgroup analysis (challenge vs non-challenge) was not conducted. In contrast, studies reporting *Salmonella* prevalence in the ceca were sub-grouped into challenge (*n* = 36) and non-challenge (*n* = 3) groups to assess the impact of colonization model. The prevalence of *Salmonella* in the ceca remained similar, regardless of mode of colonization. In the ceca, regardless of challenge route, both colonization models resulted in a similar *Salmonella* prevalence (59 %; *P* = 0.994) as shown in Fig. 3A.

For studies reporting *Salmonella* concentration in the ceca, data were categorized into three groups: challenge with a marker strain (*n* = 13), challenge with a non-marker strain (*n* = 7), and non-challenge (*n* = 5) as in Fig. 3B. The lowest *Salmonella* concentration in the ceca was observed in studies using marker strains, with an average of 3.70 log CFU (95 % CI: 2.90–4.51 log CFU). In contrast, studies using non-marker strains and natural colonization (non-challenge) exhibited significantly higher *Salmonella* concentrations at 5.77 log CFU (95 % CI: 3.74–7.81 log CFU, *P* = 0.045) and 5.16 log CFU (95 % CI: 3.39–6.93 log CFU, *P* = 0.097), respectively in the ceca.

Marker strains (e.g., *S.* Enteritidis or *S.* Typhimurium) are commonly used in *Salmonella* colonization studies, both for *in vivo* and *in vitro* studies in broilers, to enable differentiation between experimentally introduced strain and naturally occurring *Salmonella* (Cox et al., 2020). These strains are often known serotypes with high pathogenicity and induced to resist a specific antibiotic at a specific concentration. Findings from the current study indicate that challenging the birds with a marker strain results in lower cecal recovery compared to non-marker strain challenge and non-challenge models. Although all three subgroups had substantial heterogeneity with *I^2^* ≥ 99 %, trials with marker strains had a narrower CI, suggesting that their use enhances the reliability of experimental outcomes by reducing the influence of background microflora and ensuring that observed effects are attributable to the inoculated strain.

Souza et al. (2022) reported an average of 4.3 ± 1.9 log CFU/g of *Salmonella* in cecal content collected at processing plants from 45 flocks, which is between challenged with a marker strain and non-challenge models. Other studies reported cecal *Salmonella* concentrations in commercial broiler or turkey flocks are not available in literature. Alternative sampling methods such as dead-on-arrival rinsates and boot or drag swabs with approximately 3 log CFU/sample were reported and used to assess *Salmonella* load at the receiving stage of processing plants (Berghaus et al., 2013; De Villena et al., 2022; Chavez-Velado et al., 2024). Due to the limited number of studies examining *Salmonella* populations in the ceca of commercial broiler flocks, there is insufficient direct evidence to determine which colonization model best replicates commercial production conditions. However, *Salmonella* concentration reduced rapidly in chicken GIT during grow-out and is typically present at lower concentrations on broiler carcasses compared to *Campylobacter* (Stern, 2008; Berghaus et al., 2013; Wang et al., 2024). Use of marker strain for colonization during grow-out, followed by evaluating the prevalence and concentrations at processing may show evidence of the impact of *Salmonella* colonization at preharvest and translocation to the meat during poultry processing.

### Efficacy of organic acid in the crop

**Fig. 4:**
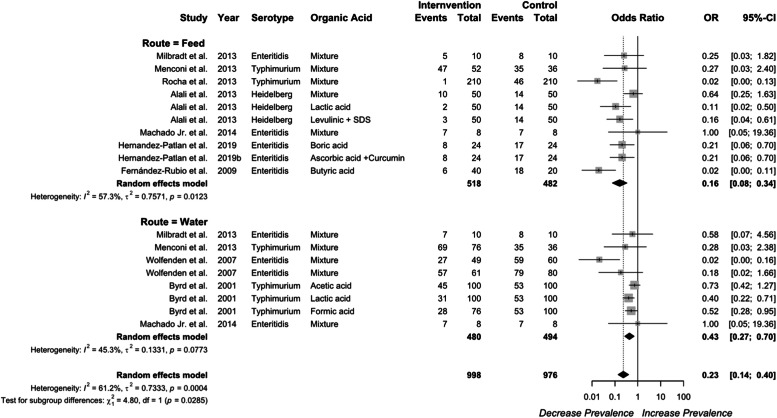
Delivery route of organic acid on *Salmonella* prevalence in the crop. Forest plot of subgroup analysis for organic acid delivery in water or feed on *Salmonella* prevalence in crop. Abbreviations: OR, odds ratio; CI, confidence interval; τ^2^, tau-squared; χ^2^, chi-squared; df, degrees of freedom; I^2^, I-squared statistic. The vertical line at the odds ratio value of one (1) in the scale of the forest plot is the line of no effect. The vertical dash line in the scale of the forest plot is the line of overall effect.

***Delivery route on the efficacy of organic acid in the crop.*** Organic acid supplementation in either feed or water reduced *Salmonella* prevalence and concentration (Fig. 4). Supplementation of organic acids in water or feed both reduced *Salmonella* prevalence in the crop, with odds ratios of 0.43 (95 % CI: 0.27–0.70) and 0.16 (95 % CI: 0.08-0.34), respectively. Moreover, organic acid supplementation via feed demonstrated a greater efficacy compared to water administration (*P* = 0.029). Significant heterogeneity was detected for organic acid supplementation via water (*I²* = 45 %, *P* = 0.077), feed (*I²* = 57 %, *P* = 0.012), and across all studies (*I²* = 61.2 %, *P* < 0.01).

**Fig. 5:**
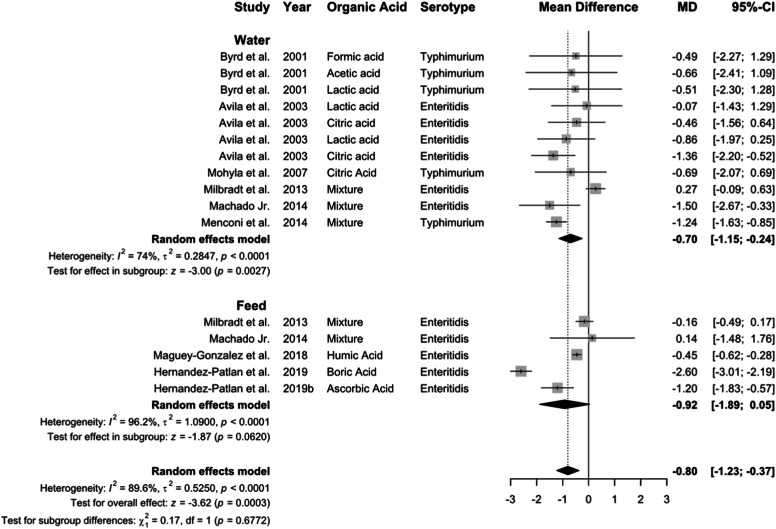
Delivery route of organic acid on *Salmonella* concentraion in the crop. Forest plot of subgroup analysis for organic acid delivery in water or feed on *Salmonella* concentration in crop. Abbreviations: MD, mean difference of log CFU/unit; CI, confidence interval; τ^2^, tau-squared; χ^2^, chi-squared; df, degrees of freedom; I^2^, I-squared statistic. The vertical line at the value of zero (0) in the scale of the forest plot is the line of no effect. The vertical dash line in the scale of the forest plot is the line of overall effect.

*almonella* concentration decreased by 0.80 log CFU, overall (95 % CI: −1.23 to −0.37; *P* < 0.01), although feed supplementation with organic acids (−0.92 log CFU) showed only numerical advantage over water (−0.70 Log CFU; Fig. 5). Significant heterogeneity was observed for organic acid supplementation via water (*I²* = 74 %, *P* < 0.01), feed (*I²* = 96 %, *P* < 0.01), and across all studies (*I²* = 90 %, *P* < 0.01).

***Mechanisms of organic acid in the crop.*** Organic acids exert bacteriostatic and bactericidal effects by lowering crop pH, inhibiting *Salmonella* growth (Byrd et al., 2001; Van Immerseel et al., 2006). Organic acids are sometimes coated with fat to allow controlled release in the gut, especially the lower intestine (Van den Borne et al., 2015). Several studies have shown that unencapsulated organic acids are more effective in killing *Salmonella* in the crop than ceca because these organic acids are most likely being absorbed in the upper gastrointestinal tract (Wolfenden et al., 2007). Current results indicate that organic acid supplementation, whether in water or feed, could reduce *Salmonella* prevalence and concentration in the crop. While supplementation of the feed was more effective than water in reducing *Salmonella* prevalence (0.16 vs. 0.42, *P* = 0.028), the reduction in *Salmonella* concentration was minimal (−0.92 vs. −0.70, *P* = 0.677). Several factors may contribute to these observations. Since the crop generally harbors lower *Salmonella* concentration compared to the lower gastrointestinal tract, the potential for substantial reductions in concentration may be inherently limited.

Broilers on *ad libitum* feeding typically consume water post-feed, accelerating liquid passage but prolonging feed contact (Rodrigues and Choct, 2018). This extends acid exposure, facilitating microbial fermentation and endogenous acid exposure (Kristoffersen et al., 2021; Abbas Hilmi et al., 2007). Additionally, water-based application often coincides with preslaughter feed withdrawal, shortening exposure time of treatment (Byrd et al., 2001). Further studies aimed at optimizing the application of organic acids to reduce *Salmonella* in the foregut are necessary to guide the poultry producers.

### Efficacy of organic acid in the ceca

**Fig. 6:**
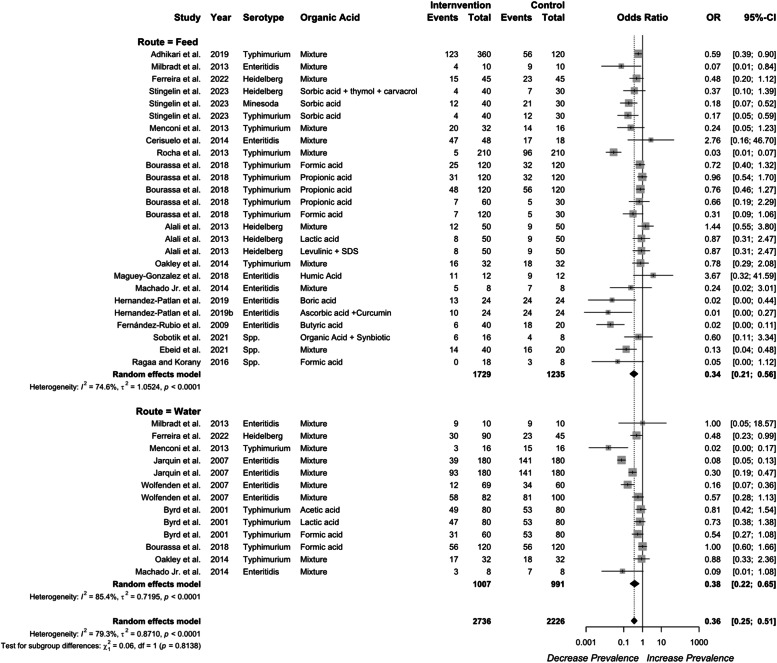
Delivery route of organic acid on *Salmonella* prevalence in the ceca. Forest plot of subgroup analysis for organic acid delivery in water or feed on *Salmonella* prevalence in ceca. Abbreviations: OR, odds ratio; CI, confidence interval; τ^2^, tau-squared; χ^2^, chi-squared; df, degrees of freedom; I^2^, I-squared statistic. The vertical line at the odds ratio value of one (1) in the scale of the forest plot is the line of no effect. The vertical dash line in the scale of the forest plot is the line of overall effect.

**Fig. 7:**
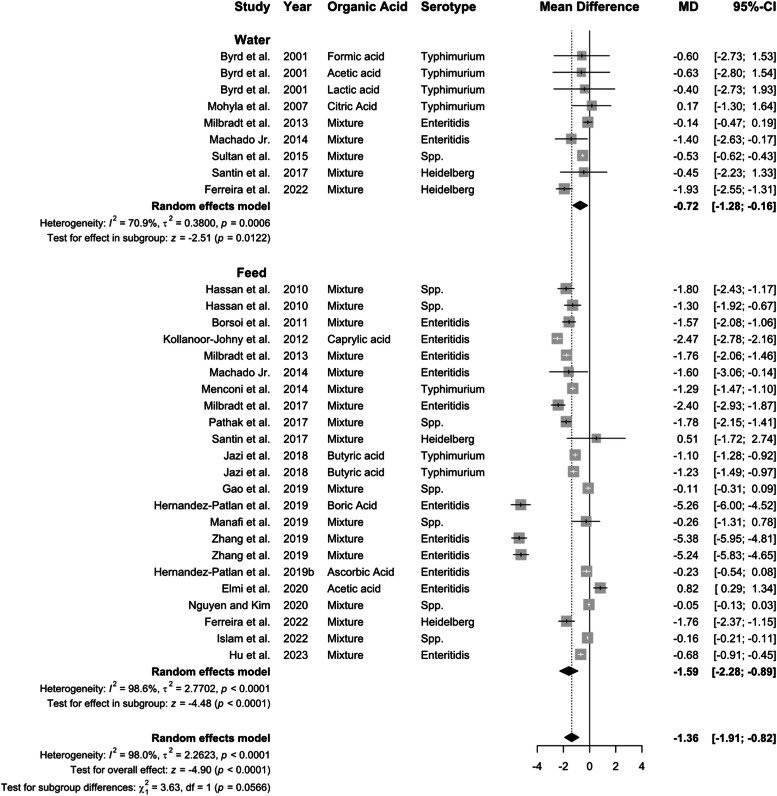
Delivery route of organic acid on *Salmonella* concentration in the ceca. Forest plot of subgroup analysis for organic acid delivery in water or feed on *Salmonella* concentration in ceca. Abbreviations: MD, mean difference of log CFU/unit; CI, confidence interval; τ^2^, tau-squared; χ^2^, chi-squared; df, degrees of freedom; I^2^, I-squared statistic. The vertical line at the value of zero (0) in the scale of the forest plot is the line of no effect. The vertical dash line in the scale of the forest plot is the line of overall effect.

***Delivery route on the efficacy of organic acid in the ceca.*** A total of 39 studies were identified that reported the effect of organic acid supplementation on *Salmonella* prevalence in the ceca, with 13 studies administering organic acids through water and 26 through feed. Overall, organic acid supplementation reduced *Salmonella* prevalence (OR: 0.36, 95 % CI: 0.25–0.51, *P* < 0.01) and concentration (−1.36 log CFU; 95 % CI: −1.91 to −0.82; Fig. 6, Fig. 7). Feed delivery (−1.59 log CFU) outperformed water (−0.72 log CFU), with high heterogeneity for all groups (*I^2^* ≥ 70.9 %, *P* < 0.01)

***Effect of organic acid on gut pH.*** Organic acids have been used as a strategy to modulate the intestinal microbiota by suppressing undesirable microbiota, including *Salmonella, Campylobacter*, and *Clostridium* (Guyard-Nicodème et al., 2016; Kumar et al., 2022; Hu et al., 2023). The current results suggested that organic acid supplementation, regardless of water and feed, reduced cecal *Salmonella* concentration and prevalence in broiler or turkey. Multiple mechanisms have been proposed in literature on the role of organic acids in reducing *Salmonella* colonization in poultry. A primary effect is reducing luminal pH (Corrier et al., 1990; Fathi et al., 2016; Martínez et al., 2021), thus creating an unfavorable environment for *Salmonella* survival and multiplication. Lowering the pH in the gastrointestinal tract limits *Salmonella* adhesion to epithelial cells and enhances the proliferation of beneficial microbes that compete with pathogens (van Immerseel et al., 2006). However, the large pH variation throughout the bird’s GIT may induce the dissociation of the organic acid, prior to the contact with pathogens at the target intestinal segment (Ma et al., 2021). The hindgut harbors most of the pathogenic bacteria and is responsible for the cross-contamination during processing (Olsen et al., 2003; Fries-Craft et al., 2024). Therefore, encapsulation of the organic acids has been used to ensure the target release of organic acids and their derivatives in the hindgut to improve the efficacy (Stefanello et al., 2020).

***Effect of organic acid on gut microbiota.*** Organic acid supplementation increases the short-chain fatty acid (SCFA) concentration in broiler ceca content and modulates gut microbiota (Aljumaah et al., 2020; Dai et al., 2021). SCFAs are metabolites from bacteria fermentation of complex non-digestible polysaccharides in the hindgut. SCFAs play a critical role in host-pathogen interactions from intestinal chemical environment modification of lowering pH through regulating and maintaining gut barrier integrity and inflammatory cytokine gene expressions (Liu et al., 2021). Research indicated that organic acid supplementation, regardless of *Salmonella* infection, increased the concentrations of butyric acid and acetic acid in broiler ceca by increasing the relative abundance of Bacteroidetes at the expense of Firmicutes (Aljumaah et al., 2020). It is known that gut microbiota and their metabolites (SCFAs) could directly or indirectly influence the host’s immune system and health (Gasaly et al., 2021). The beneficial effect in gut microbiota and SCFAs from organic acid supplementation, especially butyric acid, also enhances broiler gut barrier function by upregulating the mRNA expression of tight junction proteins, including Zonula Occludens-1, claudin, and occluding, as well as improving intestinal morphology (Yang et al., 2018; Zou et al., 2019; Aristimunha et al., 2020). Additionally, SCFA can reduce levels of lipopolysaccharides, a main component of gram-negative bacteria and stimulator of the inflammatory response, resulting in a reduction in the inflammatory response in the GIT (Jiang et al., 2015; Shang et al., 2015). These beneficial effects on gut integrity and immunity also contribute to limiting *Salmonella* and colonization of other pathogens such as *Campylobacter* and *Clostridium perfringens* (Zhen et al., 2018; Pham et al., 2020).

***Effect of organic acid on Salmonella translocation.*** A major food safety concern with *Salmonella* infection in broilers is its ability to translocate from the gastrointestinal tract to edible internal organs such as the liver. This systemic spread occurs when gut barrier function is compromised, allowing bacteria to enter the bloodstream and colonize internal tissues. While the current meta-analysis did not include *Salmonella* translocation due to the limited studies identified. However, previous studies have demonstrated that reductions in cecal *Salmonella* concentration are associated with lower prevalence in the liver and spleen (Wang et al., 2024). Given that organic acid supplementation has been shown to improve gut barrier integrity and reduce cecal *Salmonella* loads, it is plausible that these interventions could also limit bacterial translocation and help mitigate systemic infection in broilers.

***Effect of carbon chain length on organic acid efficacy.*** Van Immerseel et al. (2006) reported that medium chain fatty acids (MCFAs, C_6_ to C_12_) have a greater antibacterial activity against *Salmonella* than SCFAs (< C_6_) based on the summarized results from *in vitro* studies. MCFAs (C_6_ to C_12_) primarily exert bactericidal effects by disrupting bacterial membranes in both gram-negative and gram-positive bacteria (Nakai and Siebert, 2003). Caproic acid (C_6_) reduced *S*. Enteritidis colonization in chicks by decreasing cecal and organ invasion at 3 g/kg feed (Van Immerseel et al., 2004). Caprylic acid was also reported to reduce *Salmonella* concentration in ceca (Kollanoor-Johny et al., 2012; Manjankattil Rajan, 2020). SCFAs (< C_6_) demonstrate more bacteriostatic effects by reducing motility and biofilm formation (Lamas et al., 2019; Liu et al., 2022). However, antimicrobial efficacy of SCFAs in reducing the *Salmonella* in birds are inconsistent, especially for acetic acid (Atabaigi Elmi et al., 2020; Brizuela M, 2024). This is potentially due to acetic acid also suppressing *Lactobacillus* growth in the GIT (Van Immerseel et al., 2006). Meanwhile, acetic acid has been reported to activate SirA/BarA system then increases the virulence gene *hilA* expression in *Salmonella* (Thompson and Hinton, 1997; Lawhon et al., 2002; Pérez-Morales et al., 2021). However, propionic and butyric acids have been shown to decrease *hilA* expression for reducing the virulence of *Salmonella* and lower *Salmonella* concentrations in ceca (Van Immerseel et al., 2002). Butyric acid or blends with other SCFAs and MCFAs also have shown to increase cecal *Lactobacillus* population and improve intestinal functionality (Jazi et al., 2019; Nguyen and Kim, 2020). Additionally, propionic and butyric acids serve as energy sources for intestinal epithelial cells and maintain intestinal homeostasis (Goverse et al., 2017). Variation in the efficacy of different organic acids in reducing *Salmonella* colonization suggests that their mechanisms of action extend beyond direct antimicrobial effects. Their interactions with host physiology, gut microbiota, and *Salmonella* virulence regulation also play significant roles. Additionally, combining different organic acids was shown to have synergistic bactericidal effects *in vivo* against foodborne pathogens (Kim and Rhee, 2013; Peh et al., 2020), which leads to an increasing trend for testing organic acid mixtures recently.

### Combination with other additives

**Fig. 8:**
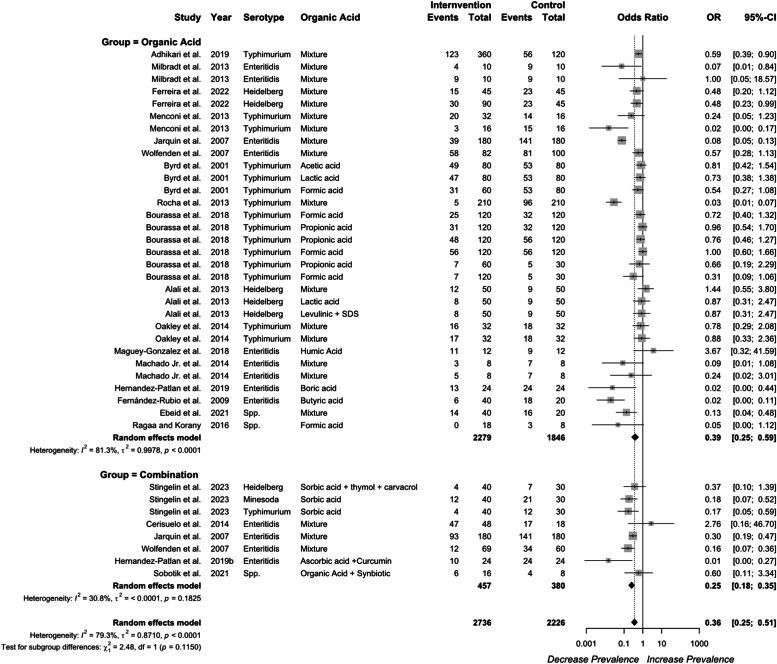
Combination effect of organic acid on *Salmonella* prevalence in ceca. Forest plot of subgroup analysis for organic acid or combination with other antibiotic alternatives on *Salmonella* prevalence in ceca. Abbreviations: OR, odds ratio; CI, confidence interval; τ^2^, tau-squared; χ^2^, chi-squared; df, degrees of freedom; I^2^, I-squared statistic. The vertical line at the odds ratio value of one (1) in the scale of the forest plot is the line of no effect. The vertical dash line in the scale of the forest plot is the line of overall effect.

**Fig. 9:**
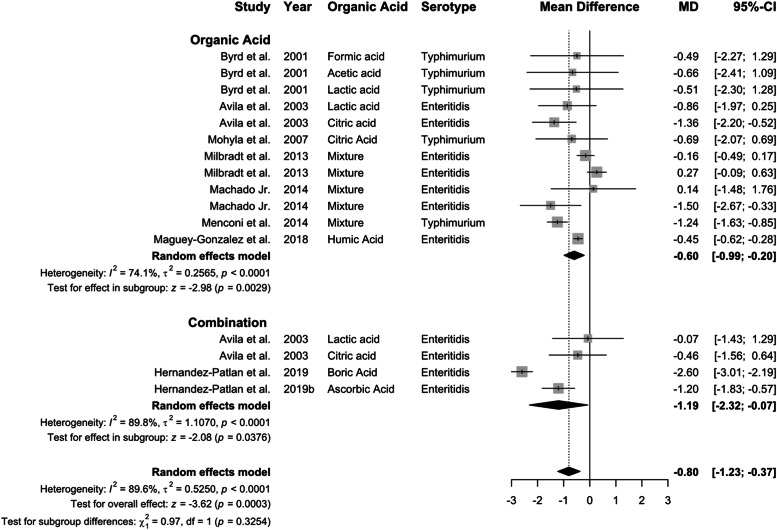
Combination effect of organic acid on *Salmonella* concentration in crop. Forest plot of subgroup analysis for organic acid or combination with other antibiotic alternatives on *Salmonella* concentration in crop. Abbreviations: MD, mean difference of log CFU; CI, confidence interval; τ^2^, tau-squared; χ^2^, chi-squared; df, degrees of freedom; I^2^, I-squared statistic. The vertical line at the value of zero (0) in the scale of the forest plot is the line of no effect. The vertical dash line in the scale of the forest plot is the line of overall effect.

**Fig. 10:**
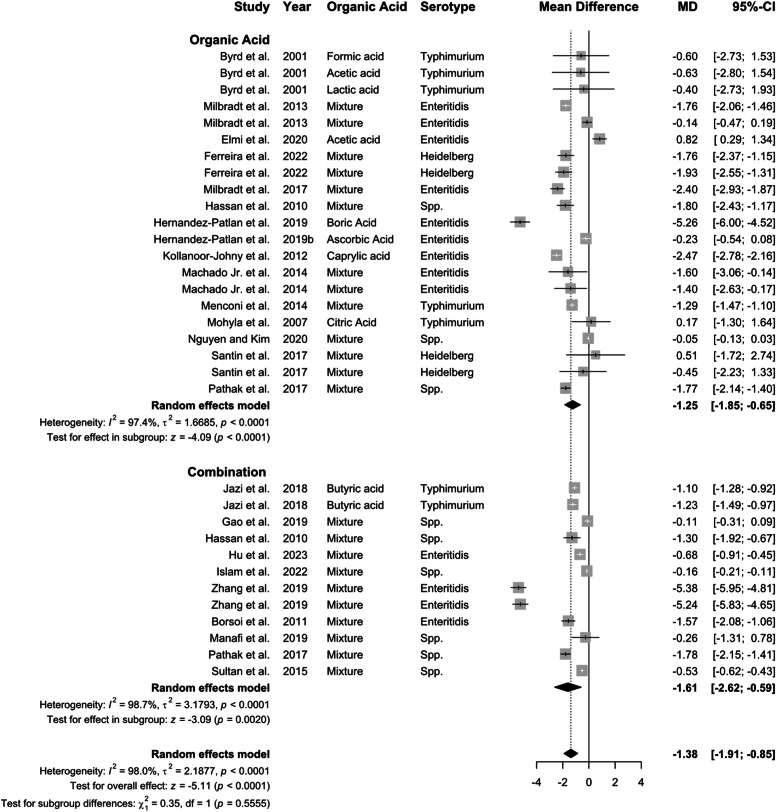
Combination effect of organic acid on *Salmonella* concentration in ceca. Forest plot of subgroup analysis for organic acid delivery in water or combination with other antibiotic alternatives. Abbreviations: MD, mean difference of log CFU; CI, confidence interval; τ^2^, tau-squared; χ^2^, chi-squared; df, degrees of freedom; I^2^, I-squared statistic. The vertical line at the value of zero (0) in the scale of the forest plot is the line of no effect. The vertical dash line in the scale of the forest plot is the line of overall effect.

***Combination effect of organic acid with other interventions.*** Organic acids are also used in combination with other interventions, including essential oils, probiotics or prebiotics for additive or synergistic effects. The combination effect of organic acid supplementation on *Salmonella* prevalence in the ceca is presented in Fig. 8. Combination of organic acid with essential oil or probiotics reduced cecal prevalence further (OR=0.25 vs. 0.39; *P* = 0.077). Combination of organic acids with other antibiotic alternatives showed a numerical advantage in reducing *Salmonella* concentrations in the ceca and crop compared to organic acid, alone (*P* > 0.1, Fig. 9, Fig. 10). Significant heterogeneity was detected in all three models of prevalence in the ceca (*I²* = 78 %, *P* < 0.001), concentration in crop (*I²* = 90 %, *P* < 0.001), and concentration in the ceca (*I²* = 98 %, *P* < 0.001).

***Mechanisms of organic acid combining with other interventions.*** The mechanisms of action of the synergistic effect depend on the substance used in combination with organic acids. Essential oils are antimicrobials with both bactericidal and bacteriostatic effects (Mayaud et al., 2008). The antimicrobial effect of essential oils primarily depends on the plant species and bioactive compounds (Ebani et al., 2019). Essential oils are commonly used in combination with organic acids since both have antimicrobial effects, with cinnamaldehyde, carvacrol, and thymol as the most popular options. Essential oils have several mechanisms of actions on both pathogen and host including reducing adhesion to intestinal epithelial cells by inhibiting Type I fimbriae (Yin et al., 2022), disrupting *Salmonella* membrane integrity by lysing the bacterial cell wall (Chauhan and Kang, 2014), and enhancing the inflammatory response by inhibiting the NF-κβ/ CASP3 pathway (Guo et al., 2024). The disturbance on the cell wall leads to an inhibition of efflux pumps, thus increasing the susceptibility of pathogens to antimicrobials (Stingelin et al., 2022). However, high doses of essential oils have been linked to reducing feed intake and *Lactobacillus* spp. population in the small intestine (Abdel-Wareth et al., 2012), hence a lower dose of essential oil (50mg/kg) and butyric acid (1000mg/kg) is reported to be more effective than the high dose combination at 100mg/kg of essential oil with 1000 mg/kg butyric acid (Cerisuelo et al., 2014). In the case of probiotics or prebiotics, the competitive exclusion, binding with pathogens, modulating gut microbiota, and immune-modulating effects are potential mechanisms of action that increase organic acids’ effectiveness (Wolfenden et al., 2007; Jazi et al., 2019).

**Fig. 11:**
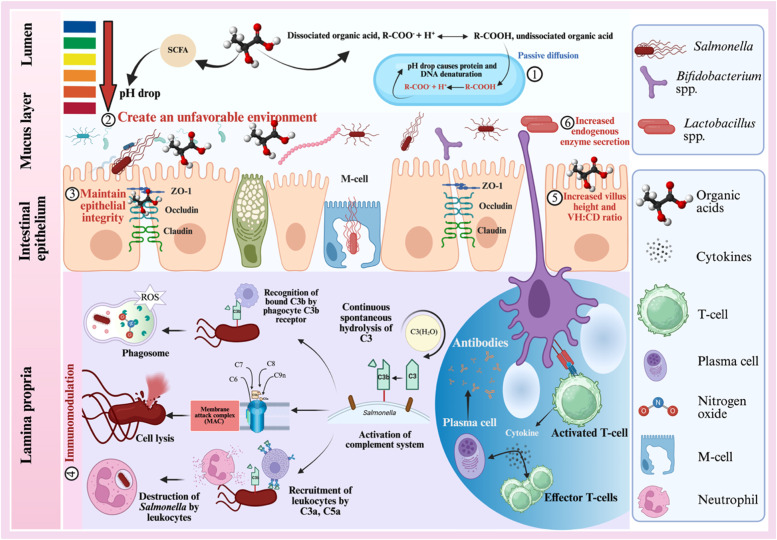
Mechanisms of organic acid supplementation reduces enteric disease colonization in the gastrointestinal tract.

The mechanisms of action against *Salmonella in vivo* and *in vitro* are well documented and elucidated in Fig. 11. Briefly, the undissociated organic acid is transported into bacterial cells by passive diffusion and causing a decrease in pH and which leads to protein and DNA denaturation and ultimately cell death (Ghazalah et al., 2011; Ángel-Isaza et al., 2019). Organic acids also lower luminal pH and create an environment less favorable to pathogenic bacteria such as *Escherichia coli, Salmonella* and *Clostridium perfringens* (Dai et al., 2021; Ma et al., 2021; Islam et al., 2024). Organic acids play a vital role in gut health and integrity, especially under an infectious status. Organic acids strengthen the intestinal barrier by regulating the expression of tight junction proteins from claudin, occludin and zona occludens, which are essential for maintaining the integrity of the epithelial layer, preventing the translocation of pathogens for systemic infection (Stefanello et al., 2020; Ma et al., 2021; Hu et al., 2023). In addition, organic acids have been shown to activate both innate and adaptive immune responses to reduce the inflammation in the intestine induced by *Salmonella* (Haq et al., 2017; Dai et al., 2021; Van Immerseel et al., 2006). Organic acids are also associated with better nutrient absorption through stimulating the secretion of endogenous enzymes and improving the intestinal morphology by improving villus height and its ratio to crypt depth (Pham et al., 2020; Hu et al., 2023). Higher villus height and lower crypt depth are beneficial to the absorption of nutrients in the intestines as it provides more surface area, thus to support a higher absorption rate for nutrients and intestinal health (Ni et al., 2012; Wang et al., 2025). The reduction of nutrient utilization due to poor intestinal morphology are associated with an increase of *Salmonella* population in the gut (Nian et al., 2011). Among all organic acids, butyric acid showed a greater effect on intestinal morphology by serving as a primary energy source of intestinal epithelial cells for supporting their function and gut barrier integrity (Salvi and Cowles, 2021). Therefore, any interventions that could potentially enhance these mechanisms including immune modulation, improving intestinal morphology and gut barrier integrity are worth exploring as part of a combined approach to optimizing pre-harvest strategies for controlling *Salmonella*.

### Potential limitations and future research

***Publication bias.*** Since significant and effective results are more often reported, this leads to an overestimation of the true effect of organic acids. The limited number of studies retrieved reduced the statistical power, such as Egger’s regression to determine publication bias (Egger et al., 1997). It is also very difficult to evaluate the true results of statistically significant publication bias tests, especially in the presence of high heterogeneity (Golden and Mishra, 2020). Nonetheless, the literature collected in the current meta-analysis and systematic review will provide perspectives on future research on developing and evaluating interventions to minimize *Salmonella* colonization at pre-harvest.

***Limitation on optimal dosage.*** A limitation of the current systematic review and meta-analysis is the inability to evaluate the dose effect of organic acid supplementation. Although some studies examined graded doses *in vivo*, the lack of detailed information on the active compounds in the intervention (types and concentrations of active compounds) prevented a dose-effect analysis using meta-regression. Additionally, including multiple dose treatments from the same study as separate data points could introduce bias by over-representing the study. To address this, we combined different dose treatments into a single index and compared them to the control group. This approach provides a more accurate estimate of the overall efficacy of organic acids. It is also important to note that some subgroups (combination group with concentration as response) only included as few as four studies. However, because meta-analysis is inherently limited in determining the optimal dosage of organic acid products, organic acid supplementation should be approached with caution. Additional research is warranted to establish the appropriate dose and confirm the safety of organic acids prior to mass implementation. While these results should be interpreted with caution, they still offer a more reliable estimate of population behavior (birds) than a single study and provide information on future research to improve the efficacy and consistency of organic acids.

***Research of pre- and post-harvest.*** Additionally, a research gap also exists on the relationship between reduction in *Salmonella* prevalence and/or concentration in the ceca at pre-harvest and the reduction of *Salmonella* prevalence and/or concentration in broiler carcasses or cut-up parts (meat) post-harvest (Edrington and Brown, 2022). Further research is necessary to disseminate the *Salmonella* transmission from poultry and poultry production environments to consumers. Given the substantial resources and efforts dedicated to pre- and post-harvest control strategies, meaningful progress is within reach.

## Conclusions

It is evident that organic acid as an antibiotic alternative could reduce *Salmonella* prevalence and concentration with both feed and water delivery, with feed delivery generally showing higher efficacy. Specifically, feed-based application has been more effective in lowering *Salmonella* prevalence in the crop and reducing bacterial concentrations in the ceca. Use of organic acids in combination with other antimicrobials such as essential oils or probiotics may enhance the antimicrobial efficacy in reducing the prevalence and/or concentration of *Salmonella* in the broiler GIT. However, caution is necessary during the delivery and blending of organic acids to ensure both safety and efficacy. Optimization of dosing, delivery methods and post-harvest impact validation remain priorities.

## CRediT authorship contribution statement

**J. Wang:** Writing – original draft, Visualization, Validation, Supervision, Software, Methodology, Investigation, Formal analysis, Data curation, Conceptualization. **B. Mallavarapu:** Writing – original draft, Validation, Investigation. **D. Subedi:** Writing – original draft, Investigation, Formal analysis. **P.S. Patil:** Writing – original draft, Investigation, Formal analysis, Data curation. **S. Bhumanapalli:** Writing – original draft, Formal analysis, Data curation. **S. Vaddu:** Writing – original draft, Formal analysis, Data curation. **A.K. Singh:** Writing – original draft, Visualization, Validation, Supervision, Methodology, Investigation, Formal analysis, Data curation, Conceptualization. **W.K. Kim:** Writing – review & editing, Supervision. **S. Kumar:** Writing – review & editing, Visualization, Validation. **S. Poudel:** Writing – review & editing, Investigation. **D.V. Bourassa:** Writing – review & editing, Validation, Supervision, Resources. **S. Manjankattil:** Writing – original draft, Visualization, Validation, Investigation. **M. Naeem:** Writing – original draft, Investigation, Formal analysis. **Y. Adhikari:** Writing – original draft, Visualization, Validation, Data curation. **H. Thippareddi:** Writing – review & editing, Supervision, Resources, Project administration, Conceptualization.

## Disclosures

This letter is to certify that the authors of this work do not have a conflict of interest or competing interests related to the work contained herein.

## Appendix Supplementary materials

Image, application 1

## Footnotes

Supplementary material associated with this article can be found, in the online version, at doi:10.1016/j.psj.2025.106075.
