# Supplementary material for: A systematic review and meta-analysis of the efficacy of organic acids in reducing Salmonella colonization in the crop and ceca of broilers
Source: Poult Sci. 2025 Nov 7;104(12):106075. doi: 10.1016/j.psj.2025.106075 (PMC12663653; doi:10.1016/j.psj.2025.106075)
Supplement: Supplementary file 1 [file mmc1.docx]

**Table S1.** Description information on organic acid component, dose, challenge age and dose, experimental condition, sampling age and type in the experiments on Salmonella prevalence in broiler and turkey.

| Organic acid component | Dose and delivery route | Serovar | Challenge age and dose | Experimental condition and species | Sampling day, and type | References |
| --- | --- | --- | --- | --- | --- | --- |
| Prevalence |  |  |  |  |  |  |
| Feed-grade buffered formic acid (61%)  and sodium formate (20.5%) mixture | 0.3, 0.6 and 0.9% in feed | *S.* Typhimurium, nalidixic acid resistant | 4 d; 1 × 10^7 CFU/mL orally | Pens; Cobb 500 | 9, 24 and 38 dpi; ceca | Adhikari et al., 2020 |
| A mixture of short- and medium-chain fatty acids (acetic (minimum 37.4 g/ kg), formic (minimum 73.6 g/kg), propionic, sorbic acid, polysorbate, coconut oil, ammonium hydroxide, vegetable fatty acid (minimum 3771 g/kg) | 2 g/kg in feed from day 1 to 90 | *S.* Enteritidis (phagotype 4), nalidixic acid and rifampicin resistant | 72 h and 24 h before slaughter; 3.2×10^9 and 4.8×10^9 CFU/bird orally | Floor pens; British Uni- ted Turkeys | Day 90; crop and caecum | Milbradt et al., 2014 |
| Organic acid blend (formic, acetic acid (minimum of 726 g/kg) , ammonium formate, mono- and diglycerides of unsaturated fatty acid, copper acetate, vegetable fatty acids (minimum of 4440 g/kg) | 1.5 g/L in drinking water for 5 days before slaughter | *S.* Enteritidis (phagotype 4), nalidixic acid and rifampicin resistant | 72 h and 24 h before slaughter; 3.2×10^9 and 4.8×10^9 CFU/bird orally | Floor pens; British Uni- ted Turkeys | Day 90; crop and caecum | Milbradt et al., 2014 |
| Formic acid and propionic acid (SCFA) or formic acid, propionic acid, caprylic acid, and capric acid (SCFA + MCFA) | 0.2 mL/L in drinking water from 6 hpi to 72 hpi | *S.* Heidelberg, nalidixic acid and spectinomycin resistant | 8 d; 0.5 mL of 10^8 CFU/mL orally | Experimental cages; commercial broilers | 24, 48, 72 hpi; cloacal swabs and cecal content | Ferreira et al., 2022 |
| Formic acid, propionic acid, caprylic acid, and capric acid (SCFA + MCFA) | 2 g/kg in feed from 6 hpi to 72 hpi | *S.* Heidelberg, nalidixic acid and spectinomycin resistant | 8 d; 0.5 mL of 10^8 CFU/mL orally | Experimental cages; commercial broilers | 24, 48, 72 hpi; cloacal swabs and cecal content | Ferreira et al., 2022 |
| Acetic acid and propionic acid blend | 0.03% and 0.06% in drinking water from day 1 to day 42 | Naturally occurring Salmonella spp. in gut (no artificial challenge) | Not challenged artificially | Floor pens; Ross 308 broilers | Day 42 (6 wk); cecal content and breast meat | Ebeid et al., 2021 |
| Sorbic acid (25%), thymol (9.5%), carvacrol (2.5%) microencapsulated in lipid matrix | 2 kg/ton from 1–21 d and 1 kg/ton from 35–42 d in feed; 2 kg/ton from 35–42 d in feed (only last wk) | S. Heidelberg*,* S. Typhimurium*,* S. Minnesota | 3 d; 1 × 10⁸ CFU/bird orally | Floor pens; commercial broilers | 7, 14, 21, 28, 35, 42 d; cecal content and liver; 6, 13, 20, 27, 34, 41 d; cloacal swabs | Stingelin et al., 2023 |
| Proprietary blend of organic acids and botanicals (includes acetic acid, propionic acid, lactic acid, citric acid, and carvacrol) | 0.1% in drinking water from 1 to 7 dpi | S. Typhimurium*,* nalidixic acid resistant | 1 d; 1 × 10⁷ CFU/bird orally | Battery cages; Cobb-Vantress broilers | 3, 7 dpi; cecal tonsils and crop | Menconi et al., 2013 |
| Sodium butyrate (1 g/kg) + EO blend (cinnamaldehyde 4.5%, thymol 13.5%) | 50 or 100 mg/kg EO + 1 g/kg Na-butyrate in feed from day 1 to 42 | S. Enteritidis*,* kanamycin resistant | 7 d; 1 × 10⁸ CFU/chick orally | Floor pens; Hubbard broilers | Feces: days 10 (72 hpi), 23, 37; Cecum and liver: day 42 | Cerisuelo et al., 2014 |
| Formic acid and propionic acid | 0.1% in drinking water from day 1 to 42 | S. Typhimurium*,* nalidixic acid resistant | 1 d; 1 × 10⁶ CFU/chick orally | Floor pens; Cobb-Vantress broilers | 3, 6, 9 dpi; crop and ceca | Jarquin et al., 2007 |
| Blend of organic acids | 1:128 dilution in drinking water continuously from day of hatch | S. Enteritidis*,* nalidixic acid resistant, phage type 13A | Day 0; 2.8 × 10⁴ CFU orally | Brooder batteries; commercial broiler chicks | 24, 48 hpi; crop and cecal tonsils | Wolfenden et al., 2007 |
| Organic acids + *Lactobacillus*-based probiotic | 1:128 dilution of organic acids in water + 0.25 mL probiotic (1.8 × 10⁷ CFU) by oral gavage on day 0 | S. Enteritidis*,* nalidixic acid resistant, phage type 13A | Day 0; 2.4 × 10⁴ CFU orally | Brooder batteries; commercial broiler chicks | 24, 48 hpi; crop and cecal tonsils | Wolfenden et al., 2007 |
| Sodium butyrate (unprotected or partially protected with vegetable fat; 40% free + 30% protected) | 0.92 g/kg in feed from day 2 to 42 | S. Enteritidis | 5 d; 1 × 10⁵ CFU orally (20% of birds) | Floor pens; Ross broilers | Cloacal swabs: 6, 9, 13, 20, 27, 34, 41 d; Crop, cecum, liver, spleen at 42 d | Fernández-Rubio et al., 2009 |
| Lactic acid (0.5%) tested alongside acetic and formic acids (all at 0.5%) | 0.5% in drinking water during 8-h feed withdrawal | *S.* Typhimurium, nalidixic acid and novobiocin resistant | Day 35 and Day 41; 1 × 10⁸ CFU orally | Floor pens; broilers (breed not specified) | Day 42 (after 8 h FW); crop and cecal contents | Byrd et al., 2001 |
| Lactic acid (food-grade, 88% purity; final 0.44%) | 0.44% in drinking water during 10–14 h feed withdrawal (on-farm and transport) | *S.* Typhimurium (naturally occurring) | Not artificially challenged | Commercial broiler flocks (breed not specified) | Pre feed withdrawal crop, post feed withdrawal crop, pre-chill carcass rinse | Byrd et al., 2001 |
| Benzoic acid (22.44%), fumaric acid (41.34%), and 2-hydroxy-4-methylthio-butanoic acid (HMTBa) (28.40%) | 0.4% in feed from day 1 to 28 | *S.* Typhimurium, nalidixic acid resistant | 1 d; 5.0 × 10² CFU/0.5 mL orally via gavage; feed inoculated with 5.0 × 10² CFU/kg from 7–14 d | Floor pens; broilers (breed not specified) | 7, 14, 21, 28 d; crop, cecum, spleen, liver, heart; liver histopathology and serum at 14 & 28 d | Rocha et al., 2013 |
| Formic acid or propionic acid | 1 or 5 kg/ton in feed from day 1 to 42 | *S.* Typhimurium, nalidixic acid resistant (seeder chicks) | Day 0; 0.1 mL of 1.8 × 10⁴ CFU orally into 3 seeder chicks per pen | Floor pens; Cobb 500 male broilers | Weekly litter; ceca wk 3 and 6 (fullfed + 12 h feed withdrawal) | Bourassa et al., 2018 |
| Formic acid or propionic acid | 1 mL/L in drinking water or 2kg/ton in feed from day 1 to 42 | *S.* Typhimurium, nalidixic acid resistant (seeder chicks) | Day 0; 0.1 mL of 2 × 10⁷ CFU orally into 2 seeder chicks per pen | Floor pens; Cobb 500 male broilers | Weekly litter; ceca wk 3 and 6 (fullfed + 12 h feed withdrawal) | Bourassa et al., 2018 |
| Formic acid or propionic acid | 4 or 6 and 5 or 10 kg/ton in feed from day 1 to 42 | *S.* Typhimurium, nalidixic acid resistant (seeder chicks) | Day 0; 0.1 mL of 1 × 10⁶ CFU orally into 2 seeder chicks per pen | Floor pens; Cobb 500 male broilers | Litter wk 3, 5, 6; ceca wk 6 (fullfed + 12 h feed withdrawal) | Bourassa et al., 2018 |
| Blend of formic, propionic, and lactic acid; lactic acid; levuliniv acid | 0.4%, 0.44%, or 0.5% in drinking water from day 0–7 and 35–42 | *S.* Heidelberg, nalidixic acid resistant | Day 1; 5 × 10⁶ CFU/0.1 mL orally into 25 seeder chicks per pen | Floor pens; Cobb × Cobb broilers | Drag swabs: day 0, 14, 42; crop and ceca: day 42 after 8 h feed withdrawal | Alali et al., 2013 |
| Formic acid, propionic acid, ammonium formate, MCFA (in water), propionic acid and MCFA (in feed) or combined | Water: 340 ppm formic acid, 250 ppm propionic acid, 200 ppm ammonium formate, 100 ppm MCFA; Feed: 480 ppm propionic acid, 1520 ppm MCFA or combined; administered from day 0 | *S.* Typhimurium, nalidixic acid resistant | Day 0; 0.1 mL of 10⁷ CFU orally into 2 seeder chicks per pen | Floor pens; Ross × Cobb broilers | Ceca at day 0, 7, 21, 42 | Oakley et al., 2014 |
| Formic acid (85%) or potassium diformate (35% formic acid + 35% formate + 30% potassium) | 0.5% in feed from day 1 to day 35 | Not challenged | Not applicable | Floor pens; Cobb 500 broilers | Day 35; ceca | Ragaa and Korany, 2016 |
| Butyric acid (coated, 30% active sodium butyrate) | 2.5 g/kg in feed from day 0 to 42 | *S.* Enteritidis phage type 4 | Day 5; 1 × 10⁵ CFU orally into 10 seeder chicks per group | Floor pens; Ross broilers | Days 6, 9, 13, 20, 27, 34, 41; cloacal swabs; Day 42; ceca | Van Immerseel et al., 2005 |
| Formic, lactic, and acetic acids (powder blend) + oregano extract | Feed (0.2%), water (0.8%), or feed (0.2%) and water (0.8%) combined | *S.* Enteritidis | Day 15; 1 × 10⁵ CFU/mL, 1 mL via gavage | Isolated rooms; Cobb 500 broilers | Day 22 and 42; crop and ceca | Machado Jr. et al., 2014 |
| Boric acid (99–100% purity, USP grade) | 0.1% in feed from day 0 to day 10 | *S.* Enteritidis, nalidixic acid and novobiocin resistant | Day 1; 1 × 10⁴ CFU orally | Brooder battery cages; Cobb-Vantress broiler chicks | Day 3 and 10; crop and ceca-cecal tonsils | Hernandez-Patlan et al., 2019a |
| Ascorbic acid (99–100% food grade) | 0.1% in feed from day 0 to 7 (prophylactic or therapeutic) | *S.* Enteritidis, nalidixic acid and novobiocin resistant | Day 6; 1 × 10⁷ CFU orally | Brooder battery cages; Cobb-Vantress male broiler chicks | Day 7; crop and ceca-cecal tonsils | Hernandez-Patlan et al., 2019b |
| Enhanced organic acid with essential oil | 500g/MT in feed from day 0 to 42 | Not challenged | Not applicable | Cobb 700 in floor pens | Day 42; ceca and carcass rinsates | Sobotik et al., 2021 |

**Table S2.** Description information on organic acid component, dose, challenge age and dose, experimental condition, sampling age and type in the experiments on Salmonella concentration in broiler and turkey.

| Organic acid component | Dose and delivery route | Serovar | Challenge age and dose | Experimental condition and species | Sampling day, and type | References |
| --- | --- | --- | --- | --- | --- | --- |
| Concentration |  |  |  |  |  |  |
| Acetic + Propionic + Citric acid | 0.031 and 0.062% in drinking water | *S*. Typhimurium, nalidixic acid resistant | 0 d; 2 × 10^5 CFU/mL orally | Pens; Cobb 500 | 24 hours; crop and ceca | Menconi et al., 2014 |
| Lactic acid or citric acid | 0.35 to 0.8% in drinking water | *S*. Enteritidis, nalidixic acid resistant | 36 d; ~10^9 CFU/mL orally, twice | Cages with wood shavings; Broilers | 8 hours pre-slaughter; crop | Avila et al., 2003 |
| Lactic acid | 0.44 or 0.5% in drinking water | *Salmonella* Typhimurium, nalidixic acid resistant | Day 35 and Day 41; 10^8 CFU/mL orally | Floor pens; 5-wk broilers | 8 hours pre-slaughter; crop and ceca | Byrd et al., 2001 |
| Formic acid + Acetic acid + others | 1.5 g/L in drinking water | *S*. Enteritidis, nalidixic acid and rifampicin resistant | 72 and 24 h before slaughter; 3.2 × 10^9 & 4.8 × 10^9 CFU per bird | Floor pens; commercial turkeys | At slaughter; crop and cecum | Milbradt et al., 2013 |
| Formic acid + Acetic acid + Sorbic acid | Powder mix in feed; dosage not directly in % but includes min. 37.4 g/kg acetic acid | *S*. Enteritidis, nalidixic acid and rifampicin resistant | 72 and 24 h before slaughter; 3.2 × 10^9 & 4.8 × 10^9 CFU per bird | Floor pens; commercial turkeys | At slaughter; crop and cecum | Milbradt et al., 2013 |
| Formic acid + Acetic acid + Sorbic acid | 2 g/kg in feed | *S*. Enteritidis, nalidixic acid and rifampicin resistant | 18 days old; 3.8 × 10^8 CFU/bird orally | Cages; commercial turkeys (BUT Big 9) | Post-challenge days 3 and 10; cecal content | Milbradt et al., 2017 |
| Acidifier A (formic, propionic, acetic, citric acids + salts) or acidifier B (formic, propionic acids + other additives) | 3000 mg/kg in feed | *S*. Enteritidis (RITCC 1695) | 13 days; ~10^8 CFU orally | Broiler chickens (Ross 308); floor pens | Post-challenge sampling at day 42; various gut samples | Hassan Saleh et al., 2025 |
| Boric acid (BA) | 0.1% in feed | *S*. Enteritidis, novobiocin and nalidixic acid resistant | 1-day-old chicks; 10^4 CFU orally | Battery cages; Cobb-Vantress broilers | Days 3 and 10 post-challenge; crop and ceca | Hernandez-Patlan et al., 2019 |
| Ascorbic acid (AA) | 0.1% in feed | *S*. Enteritidis | 6-day-old chicks; 10^7 CFU per bird | Battery cages; broiler chickens | 24 h post-challenge and day 10; crop and cecal tonsils | Hernandez-Patlan et al., 2019b |
| Essential Oils + Organic Acids (EOA blend) | 300 to 800 mg/kg in feed | *S*. Enteritidis (CVCC3379) | 13 days; 1 × 10^9 CFU/mL orally | Arbor Acres broilers; cage housing | 3 and 10 days post-infection; cecum, liver, spleen | Hu et al., 2023 |
| Organic Acid Blend (propionic, formic, HMTBa) | 200 mg/kg in feed | Natural challenge (environmental); *Salmonella* spp. | Not applicable (field condition) | Broiler chickens (Cobb 500); floor pens | Days 21 and 35; gut microbiota and ileum | Islam et al., 2022 |
| Thymol + Benzoic acid or Cinnamylaldehyde + Caproic acid | 800 g/ton of feed | *Salmonella* Enteritidis | 7 days; 4.4 × 10^9 CFU/mL orally | SPF Leghorn chicks; wire cages | Days 3, 5, 7 post-infection; cecum, spleen, liver | Zhang et al., 2019 |
| Caprylic acid or caprylic acid | 0.7% or 1% in feed (last 5 days) | *S*. Enteritidis (4-strain mix) | Day 5 and 25; 8 log10 CFU orally | Broiler chickens; floor pens | Post-challenge days 10 and 30; intestinal and internal organs | Kollanoor-Johny et al., 2012 |
| OA + Oregano Extract (feed, water or combined) | 0.2% feed, 0.08% water or combined | *S*. Enteritidis | Day 15 post-hatch; 1 × 10^5 CFU/bird | Broilers (Cobb); 3-phase diet | Days 17, 22, 28, 42; crop and ceca | Machado Jr. et al., 2014 |
| Humic Acid (HA) | 0.1% in feed (in vitro), 0.2% in feed (in vivo) | *S*. Enteritidis, nalidixic acid and novobiocin resistant | 10 d old; 10^6 CFU/chick | Broiler chicks (Cobb); in vitro and in vivo design | 24 h post-infection; Ceca and intestinal assays | Maguey-Gonzalez et al., 2018 |
| Novacid (Propionic, Fumaric, Lactic acids + glucomannan + phytochemicals) | 0.05% in feed | Natural infection and *E. coli* challenge (not *Salmonella*-specific) | 10 d old; 10^8 CFU/mL E. coli | Broilers (Ross × Ross 308); floor pens | Day 42; cecal *Salmonella* count | Manafi et al., 2019 |
| Citric acid + Propionic acid + Essential oils | 0.05% in feed | *S*. Enteritidis (rifampicin resistant) | Day 1; 10^4 CFU/chick | Broilers (Ross 308); battery cages | Days 3 and 10 post-challenge; cecal tonsils | Menconi et al., 2013 |
| Acidified Sodium Chlorite (ASC, with Citric acid) | 600 ppm SC + CA in drinking water | *S*. Typhimurium, nalidixic acid resistant | 35 days; 10^5 CFU/bird | Broilers; Petersime battery cages and floor pens | 24 h treatment; digestive tract segments (crop to ceca) | Mohyla et al., 2007 |
| Protected OA + MCFA (fumaric, citric, malic acids + capric/caprylic acids) | 0.25 to 1 g/kg in feed | Natural condition (no explicit *Salmonella* challenge) | Not challenged | Broilers (Ross 308); 7-week floor trial | Day 49; excreta microflora and digestibility | Nguyen & Kim, 2020 |
| Formic acid + Propionic acid (0.05% in water); Fumaric + Benzoic acids (0.3% in feed); Coated Calcium Butyrate (0.2% in feed) | 0.05% water, 0.3% or 0.2% feed depending on trial | *S*. Heidelberg (UFPR1 strain) | 1 or 7 days; 10^7 CFU/bird | Broilers; 21-day cage trials in isolated rooms | Days 7, 14, 21; cecum and liver | Santin et al., 2017 |
| Aciflex® (Citric acid, Lactic acid, Phosphoric acid, CuSO4) | 0, 0.5, 1, 1.5, and 2% in drinking water | Natural infection; *Salmonella* spp. (not specified) | Natural occurrence (22–42 days) | Broilers (Hubbard); open-sided pens | Day 42; ileal digesta and microbial enumeration | Sultan et al., 2015 |
| Butyric acid (encapsulated, 0.05%) alone and in combination | 0.05% in feed | *S*. Typhimurium (10^5 CFU) | Day 3; 0.5 mL of 10^5 CFU orally | Ross 308 broilers; 24-day trial with 6 treatments | Days 7, 14, 21 post-challenge; ceca, jejunum | Jazi et al., 2018 |
| Acetic acid (AcA), 0.5%; combo with L. acidophilus and Thymus vulgaris extract | 0.5% in drinking water | *S*. Enteritidis (Nalidixic acid resistant) | Day 3; 5 × 10^9 CFU/chick | Broilers; 7 treatments, 420 birds | Days 8, 15, 24; feces, cecum, ileum | Elmi et al., 2020 |
| Encapsulated OA + EO (citric, sorbic acid + thymol, vanillin) | 150, 200, or 250 mg/kg in feed | Natural infection; *Salmonella* spp. and *E. coli* monitored | Natural; no deliberate challenge | Broilers; 70-day trial | Days 21 and 70; cecal microflora, jejunum, ileum | Gao et al., 2019 |
| Not applicable (comparison only study; no OA treatment) | N/A | *S*. Heidelberg and *S*. Enteritidis (ATCC 1980) | Day 0; 10^5 CFU/mL orally | Broiler chicks; battery cages | 6h, 12h, 72h, 21 dpi; liver, cecum, cloacal swabs | Borsoi et al., 2011 |
| Formic acid and propionic acid (SCFA) or formic acid, propionic acid, caprylic acid, and capric acid (SCFA + MCFA) | 0.2 mL/L in drinking water from 6 hpi to 72 hpi | *S*. Heidelberg (resistant to nalidixic acid and spectinomycin) | 8 days; 10^8 CFU/mL orally | Broilers; 36 birds/group | 24h, 48h, 72h post-infection; cecum, cloacal swabs | Ferreira et al., 2022 |
| Formic acid, propionic acid, caprylic acid, and capric acid (SCFA + MCFA) | 2 g/kg in feed from 6 hpi to 72 hpi | *S*. Heidelberg (resistant to nalidixic acid and spectinomycin) | 8 days; 10^8 CFU/mL orally | Broilers; 36 birds/group | 24h, 48h, 72h post-infection; cecum, cloacal swabs | Ferreira et al., 2022 |
